# Study of Zoonotic Pathogens in Alien Population of Veiled Chameleons (*Chamaeleo calyptratus*) in the Canary Islands (Spain)

**DOI:** 10.3390/ani13142288

**Published:** 2023-07-12

**Authors:** Román Pino-Vera, Néstor Abreu-Acosta, Pilar Foronda

**Affiliations:** 1 Instituto Universitario de Enfermedades Tropicales y Salud Pública de Canarias, Universidad de La Laguna, 38200 San Cristóbal de La Laguna, Spain; rpinover@ull.edu.es (R.P.-V.); gerencia@nertalab.es (N.A.-A.); 2 Department Obstetricia y Ginecología, Pediatría, Medicina Preventiva y Salud Pública, Toxicología, Medicina Legal y Forense y Parasitología, Universidad de La Laguna, 38200 San Cristóbal de La Laguna, Spain; 3 Programa de Doctorado Ciencias Médicas y Farmacéuticas, Desarrollo y Calidad de Vida, Universidad de La Laguna, Avda. Astrofísico F. Sánchez, s/n, 38203 San Cristóbal de La Laguna, Spain; 4 Nertalab S.L.U., 38001 Santa Cruz de Tenerife, Spain

**Keywords:** Canary Islands, veiled chameleons, zoonotic bacteria, *Yersinia enterocolitica*, *Salmonella*

## Abstract

**Simple Summary:**

Veiled chameleons are native to the Arabian Peninsula, with a presence in other regions, such as the Canary Islands (Spain). The aim of this study is to analyze the existence of pathogenic bacteria in a population of this invasive reptile on Gran Canaria island. The results obtained highlight the presence of a variety of pathogens with relevance to human health, most of them related to gastrointestinal diseases. This archipelago is a biodiversity hotspot, with some endangered species living there, so the presence of veiled chameleons could be also a risk to biodiversity conservation, by the spread and/or transmission of pathogenic bacteria to the native fauna. In conclusion, the invasive veiled chameleon population in the Canary Islands should be considered as a potential risk factor for biodiversity conservation and human health.

**Abstract:**

Veiled chameleons (*Chamaeleo calyptratus*) are native to the Arabian Peninsula that have been introduced as pets in many regions around the world, such as the Canary Islands (Spain). In this work, the gastrointestinal content from veiled chameleons of Gran Canaria island (Canary Islands) has been analyzed to determine the presence of zoonotic bacteria. Forty animals were analyzed using different selective culture media and PCR. The most isolated bacteria were *Yersinia enterocolitica* (52.4%), followed by *Salmonella* spp. (40.0%), with positive isolates for *Salmonella* Tyhpi and *Salmonella* Typhimurium. *Pseudomonas* spp. was found in 32.5% of the chameleons. More than half were positive for *Pseudomonas aeruginosa*. Antibiotic-resistant *Staphylococcus* spp. was detected in six animals plus one isolate of non-resistant *Staphylococcus hominis*. Multiple mycobacteria species belonging to both tuberculous and non-tuberculous complexes were identified as well as *Escherichia coli* carrying the *stx*_1_ and *eae* virulence genes with 12.5% and 7.5% prevalence, respectively. *Listeria monocytogenes*, *Campylobacter* spp., and *Vibrio* spp. were found in lower proportion (<5%). The results obtained indicate that veiled chameleons in Gran Canaria could be playing a role in the maintenance and dissemination of the pathogens detected, harming public health and biodiversity.

## 1. Introduction

The settlement of animals into new ecosystems leads to a multi-factor problem: crop loss, predation, or resource competition with the endemic species (endangered in many cases) [1] or, concerning this study, being a source of pathogens to humans, animals, and plants [1]. This last fact is especially dangerous in the case of uncommon pathogens because the local health system may not have the means and experience needed for its diagnosis and treatment [2].

The Canary Islands (Spain) are located in Norwest Africa, near the coast of Morocco (13°23′–18°80′ W and 27°37′–29°24′ N), and they present some features that make them suitable for the colonization of invasive species such as good climatic conditions, resource abundance, and lack of large predators [3]. 

All this, added to the high number of endemic species present in the Canary Archipelago, makes the introduction of exotic animals a risk to the local biodiversity [4]. At least 1167 exotic plant and animal species have been reported in the Canaries, of which 289 are considered invasive or potentially invasive species [5]. On Gran Canaria, the second largest island, the negative effect of exotic reptiles is well known, just as in the case of the California kingsnake (*Lampropeltis californiae*), which has alarmingly reduced the population of endemic lizards [6]. Apart from these snakes, the veiled chameleon, *Chamaeleo calyptratus* Duméril and Bibron, 1851 (Squamata, Chamaeleonidae), native to the southwest Arabian Peninsula, was introduced to the north of the island with available records reporting their entrance in 2017, firstly introduced as a pet and zoological species [7]. They are a diurnal and arboreal lizard species capable of adapting to a huge variety of ecosystems from high, dry plateaus to forests and river valleys. They have been also reported in Florida and Hawaii (USA) due to the illegal pet trade [8].

In the Canary Islands, the effect of these chameleons on the endemic invertebrates is known only because of their insect-based diet [7], but no data are available about the possible role in the transmission and maintenance of pathogenic agents. Due to this lack of information, the aim of this study was to determine the presence of pathogenic bacteria in these animals from Gran Canaria and evaluate the health risks to humans and local fauna. 

## 2. Materials and Methods

**Figure 1:**
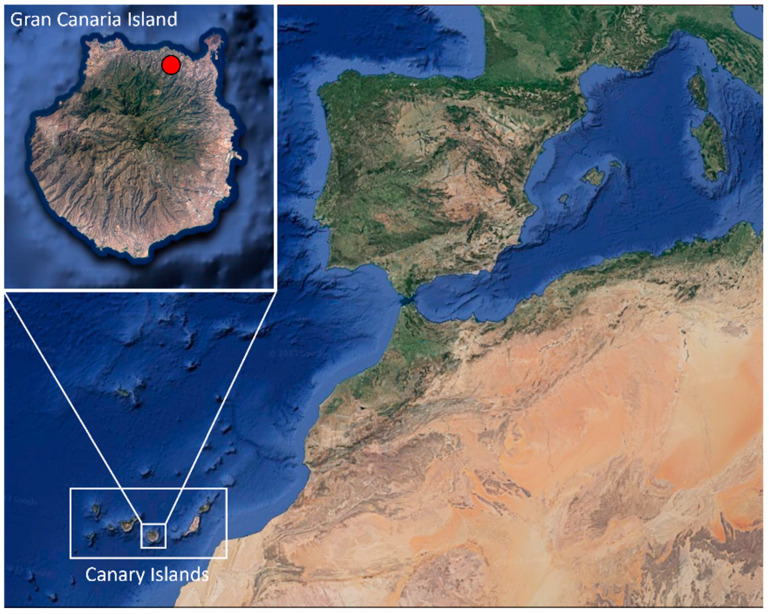
Location where *Chamaleo calyptratus* were captured (red spot) on Gran Canaria (Canary Islands, Spain). Images obtained from Google Earth website and modified with Microsoft PowerPoint 2016 software.

A total of 40 animals, 36 adults (11 males, 21 females, and 4 indeterminate) and 4 young individuals, from Arucas municipality, Gran Canaria (Canary Islands, Spain) (Figure 1), were donated by “Red de Alerta Temprana de Canarias para la Detección e Intervención de Especies Exóticas Invasoras” (REDEXOS) staff, after authorization of “Dirección General de Lucha Contra el Cambio Climático y Medio Ambiente” (Gobierno de Canarias, Expte. EEI-001/2016). The reptiles were sexed and necropsied. During that time, fecal matter was obtained for culture. 

### 2.1. Bacterial Strains

The bacterial strains used as positive controls for the assays were obtained from American Type Culture Collection (ATCC). All of them were stored at −70 °C, and incubated to make them grow for 18 to 24 h in Tryptic Soy Broth (TSB) (Labkem, Barcelona, Spain) at 37 °C under aerobic conditions, or microaerophilic conditions in the case of *Campylobacter* spp.

### 2.2. Isolation of Bacteria from the Samples

During dissections, 100 mg of intestinal content was extracted from each animal and incubated in 5 mL of Buffered Peptone Water (BPW) (Labkem, Barcelona, Spain) at 37 °C for 24 h. For the isolation of *Campylobacter* spp., 100 mg of intestinal content was incubated in 2 mL of BPW at 42 °C for 18 h under microaerophilic conditions while, in the case of *Vibrio* spp. isolation, Alkaline Peptone Water (APW) was applied instead of BPW for 8 h at 37 °C. APW was made in the laboratory from BPW, adding 1% of NaCl and increasing the pH level to 8.4.

After this first incubation, different selective culture media were used, 100 µL of peptone water culture was incubated in Baird–Parker agar (Labkem, Barcelona, Spain) for the isolation of *Staphylococcus* spp., Cetrimide agar (VWR International, Leuven, Belgium) for *Pseudomonas* spp., Cefsulodin Irgasan Novobiocin agar (CIN) (Merck, Darmstadt, Germany) for *Yersinia* spp., Sorbitol supplemented MacConkey agar (Scharlab, Barcelona, Spain), and Tryptone Bile X-glucuronide chromogenic agar (TBX) (Labkem, Barcelona, Spain) for *Escherichia coli* and Oxford agar (Labkem, Barcelona, Spain) for *Listeria monocytogenes*. For *Vibrio* spp. isolation, 100 µL of APW culture was incubated in Thiosulfate-Citrate-Bile-Saccharose agar (TCBS) (VWR International, Leuven, Belgium). All the cultures were incubated for 24 h at 37 °C except for CIN, which was incubated at 30 °C.

In the case of *Salmonella* spp., 500 µL of BPW culture was transferred to 4.5 mL of Rappaport–Vassiliadis Broth (VWR International, Leuven, Belgium) and stored for 20 h at 42 °C. Then, 100 µL of broth culture was later incubated in *Salmonella*–*Shigella* agar (Merck, Darmstadt, Germany) for 24 h at 37 °C.

### 2.3. Molecular Identification 

#### 2.3.1. DNA Extraction 

For the DNA isolation of *Mycobacterium* spp., 1 mL of each BPW culture was taken, and for *Campylobacter* spp., 1 mL of BPW cultured at 42 °C under microaerophilic conditions was used. All samples were washed twice with phosphate-buffered saline (PBS) and the pellet was subjected to DNA extraction method following López et al. indications [9]. 

For the rest of bacteria, 5 colonies of each agar plate were suspended in 1 mL of PBS and centrifuged at 12,000× *g* twice. The pellet was subjected to DNA extraction following López et al. [9].

#### 2.3.2. PCR Identification

After the DNA extraction, the most relevant zoonotic bacteria, including resistance and virulence genes, were identified using PCR techniques. All assays were performed using positive and negative controls.

For *Campylobacter* identification, a multiplex PCR (m-PCR) with six pairs of primers was employed for genus confirmation and identification of *Campylobacter coli*, *Campylobacter fetus*, *Campylobacter jejuni*, *Campylobacter lari*, and *Campylobacter upsaliensis* according to Wang et al. [10].

In the case of *E. coli*, virulence genes were analyzed: *stx*_1_ and *stx*_2_ genes, responsible for Shiga-like toxins synthesis, and *eae* gene, which codifies for intimin, following the protocol described by Blanco et al. [11].

*Listeria monocytogenes* was identified through the confirmation of the suspicious colonies grown in Oxford agar, by a simple PCR of a region of the *iap* gene, which codifies the p60 invasion-associated protein, as described by Jaton et al. [12].

Following the process described by Kim et al. [13], m-PCR was carried out for mycobacteria identification and to differentiate the *Mycobacterium tuberculosis* complex from the atypical mycobacteria group. 

UV fluorescent colonies in Cetrimide agar were tested for *Pseudomonas aeruginosa* through simultaneous amplification of *oprI* and *oprL* genes, which codify for lipoproteins, as described by De Vos et al. [14].

Regarding the determination of *Salmonella* serotypes important to human health, Guimarães de Freitas et al. [15] protocol was followed. One m-PCR allows the detection of all bacteria belonging to the genus, and the identification of *Salmonella* Enteritidis and *Salmonella* Typhi serotypes. A second PCR was utilized to identify *Salmonella* Typhimurium serotype.

A single m-PCR was used for the identification of *Staphylococcus* aureus, *Staphylococcus epidermidis*, *Staphylococcus haemolyticus*, *Staphylococcus hominis*, *Staphylococcus lugdunensis*, and *Staphylococcus saprophyticus* species as well as the detection of antibiotic-resistant genes (methicillin and mupirocin) following Campos-Peña et al. [16].

For the most frequent *Vibrio* species, a PCR assay was performed to detect the genus according to Liu et al. [17]. The positive samples were subjected to more specific PCR to identify *Vibrio cholerae*, *Vibrio parahaemolyticus*, and *Vibrio vulnificus*, as described by Neogi et al. [18].

In the case of *Yersinia enterocolitica*, two pairs of primers were used to detect pathogenic and non-pathogenic strains, according to Wannet et al. [19].

The results of all PCR assays were evaluated with agarose gel electrophoresis (Fisher Bioreagents, Madrid, Spain), and the size of the amplification products was estimated by comparison with molecular size markers (SiZer-100 DNA Marker, iNtRON Biotechnology, Seongnam-Si, Republic of Korea; and AmpliSize Molecular Ruler, Bio-Rad, Hercules, CA, USA). Real-Safe (Durviz SL, Valencia, Spain) was used as a DNA stain and a ChemiDocTM XRS+ (Bio-Rad, Hercules, CA, USA) system for the visualization of the amplicons. 

### 2.4. Co-Infection Index (Ic)

The co-infection index (Ic) developed by Ginsberg [20] quantifies the deviation of the number of mixed infections from independence. It is defined as the difference between the number of co-infections and the expected number due to chance alone, as a percentage of the totality of the infected animals.

Ic = [(O − E)/N] × 100

where O = number of observed co-infections; E = expected number of *C. calyptratus* with coinfections due to chance alone; N = total number of *C. calyptratus* infected by either or both microorganisms.

E = (a + b) (a + c)/(a + b + c + d); N = a + b + c

where a = number of chameleons infected by both bacteria (equals O); b = number of chameleons infected only with microorganism 1; c = number of chameleons infected only with microorganism 2; and d = number of chameleons not infected with microorganism 1 nor 2. The Ic value is positive if the number of real co-infections is greater than that expected or negative if it is less. The significance of the co-infection index was calculated by chi-square test.

### 2.5. Statistical Analysis

The statistical Windows software “SPSS” version 25.0 (IBM Corporation, Armonk, NY, USA) was used to compare the prevalence between sex and age of the studied individuals. For that purpose, chi-square test and Fisher’s exact test were applied with a stabled *p*-value of 0.05.

## 3. Results

Twenty-eight out of forty studied animals (70.0%) showed positive results for at least one of the investigated bacteria. The most frequent pathogen was *Y. enterocolitica*, confirmed in 52.4% of the isolates, followed by *Salmonella* spp. (40.0%) and *Pseudomonas* spp. (32.5%). Table 1 describes all positive isolates.

### 3.1. Campylobacter spp.

Among the five *Campylobacter* species sought, just *C. lari* was identified in a male chameleon, and a female was positive for a *Campylobacter* species that was not included in the assays. None of the young individuals were infected with this group of bacteria.

### 3.2. Escherichia coli Virulence Genes (stx1, stx2, and eae)

*Escherichia coli* containing virulence genes was identified in three (7.5%) chameleons, two individuals carrying the *stx*_1_ gene and another one carrying the *eae* gene. The three animals were females, not being found in males or young individuals. The *stx*_2_ gene was not identified, nor was the coexistence of *E. coli* with more than one gene in the same animal.

### 3.3. Listeria monocytogenes

The *iap* gene amplification allowed the identification of two females positive for *L. monocytogenes*. None of the males or young individuals were infected with this bacterium.

### 3.4. Mycobacterium spp.

Five chameleons (12.5%) were positive for *Mycobacterium* spp. The amplification only of the *rpoB* gene in three of them (7.5%) indicates an infection with *Mycobacterium microti*, included within the tuberculous complex, or with a non-tuberculous mycobacterium. In the other two cases, the amplification of *RD1* and *rpoB* genes suggests a *Mycobacterium bovis* infection, a tuberculous mycobacterium. There were no significant differences between infected males and females or adult and young individuals (*p* > 0.05). 

### 3.5. Pseudomonas spp.

**Figure 2:**
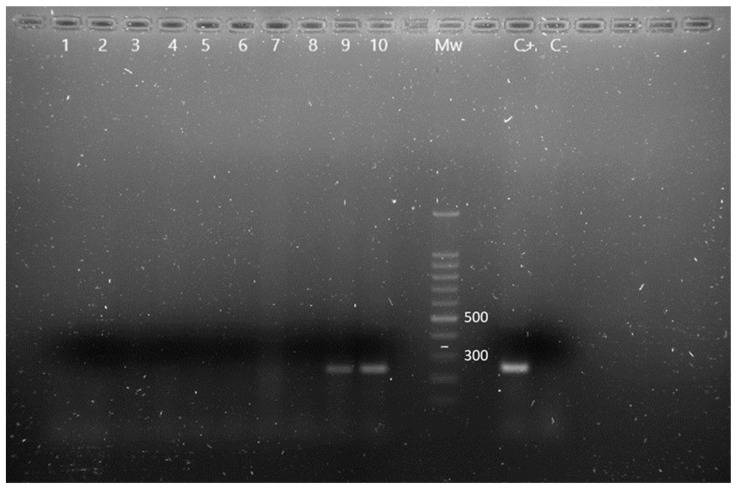
m-PCR results for the detection of *Pseudomonas* spp. and *P. aeruginosa* in *Chamaeleo calyptratus* from Gran Canaria (Canary Islands, Spain). Lane 1 to 8: negative samples for *Pseudomonas* spp., lane 9 and 10: *OprI* gen amplification fragments characteristic of *Pseudomonas* spp., Mw: molecular size marker (SiZer-100 DNA Marker, iNtRON Biotechnology), C+: positive control, C−: negative control.

Thirteen animals were positive for *Pseudomonas* spp., being identified *P. aeruginosa* in eight of them, by simultaneous *oprI* and *oprL* gene amplifications (Figure 2). There were no significant prevalence differences between males and females or adult and young individuals (*p* > 0.05). The obtained results, classified according to age and sex, are described in Table 2.

### 3.6. Salmonella spp.

**Figure 3:**
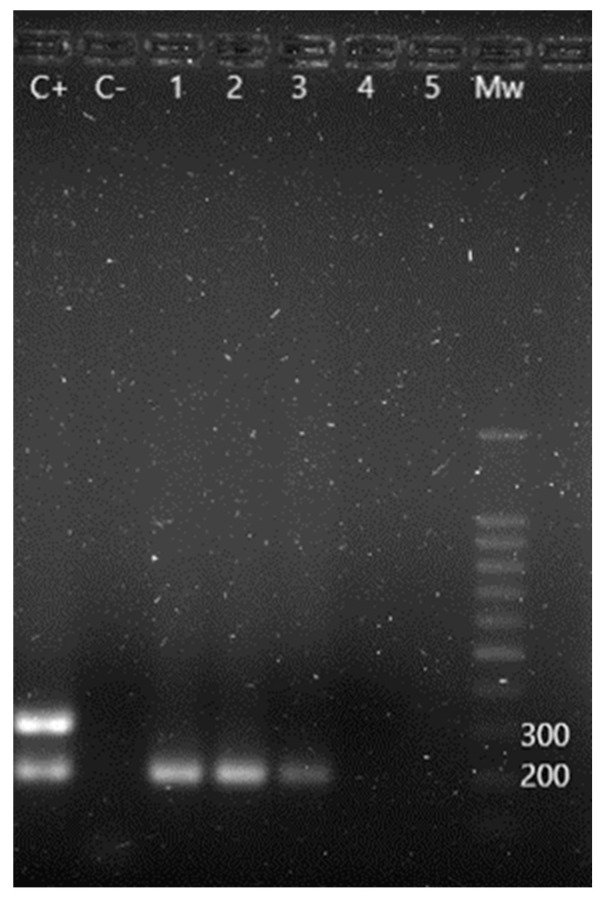
m-PCR results for the detection of *Salmonella* spp. and its serotypes. C+: *S.* Enteritidis positive control showing a 204 bp amplification fragment corresponding to *Salmonella* spp. and a 304 bp fragment corresponding to *S.* Enteritidis serotype. C−: negative control. Lane 1 to 3: positive samples for *Salmonella* spp. Lane 4 and 5: negative samples. Mw: molecular size marker (SiZer-100 DNA Marker, iNtRON Biotechnology).

Sixteen out of forty animals (40.0%) presented positive results for *Salmonella* spp., having found *S.* Typhi in two of them and *S.* Typhimurium in another two, apart from one case of coinfection with both serotypes (Figure 3). *Salmonella* Enteritidis was not detected. No significant difference prevalences were found, according to sex and age. Table 3 shows the results obtained regarding this bacteria genus.

### 3.7. Staphylococcus spp.

Among the six *Staphylococcus* species searched, just *S. hominis* was found in one of the studied animals (2.5%). Despite this, multiple *Staphylococcus* spp. carrying antibiotic-resistant genes that could not be identified at the species level were detected, including one isolate with both genes. Statistical tests showed no significant differences between males and females or between adults and juveniles individuals. These results are described in Table 4.

### 3.8. Vibrio sp.

*Vibrio* sp. was only found in a female chameleon. One PCR assay performed after the genus confirmation showed a negative result, indicating a species different from *V. vulnificus*, *V. parahemolyticus*, and *V. cholerae*.

### 3.9. Yersinia enterocolitica

**Figure 4:**
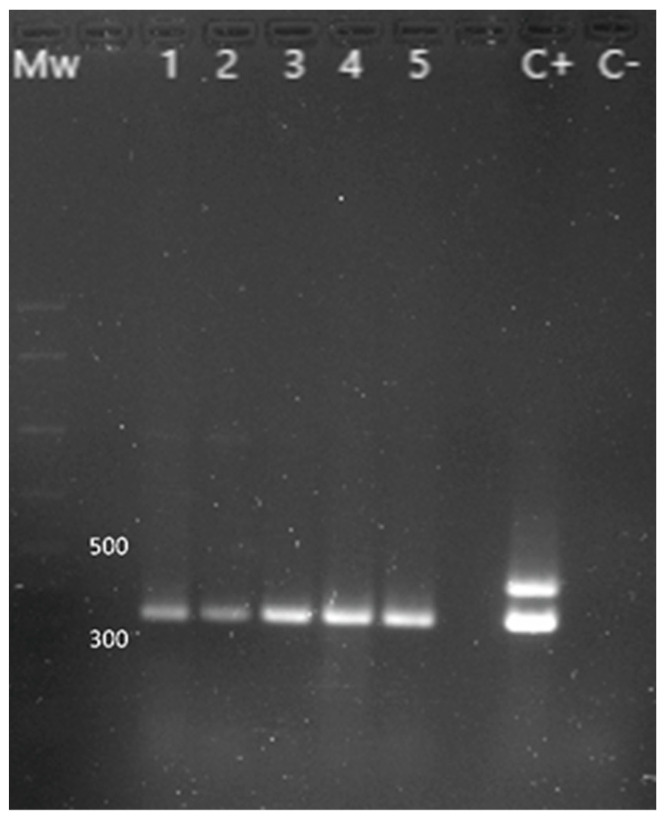
PCR results for the identification of *Y. enterocolitica*. Mw: molecular size marker (AmpliSize Molecular Ruler, Bio-Rad). Lane 1 to 5: 16s gene (330 bp) amplification fragment characteristic of *Y. enterocolitica*. C+: positive control (the 425 bp fragment corresponding to the *ail* gene is only found in pathogenic strains). C−: negative control.

The presence of *Y. enterocolitica* was investigated in 21 chameleons (Figure 4), with 11 positive animals (52.4%): 7 out of 11 females (63.6% of the females studied), 2 males out of 6 (33.3% of the males studied), and 2 out of 4 juveniles (50.0% of the juveniles studied). The *ail* gene amplification, characteristic of pathogenic strains, was only observed in one isolate. Statistical tests did not expose significant differences when comparing the prevalence between sex and age of the individuals.

### 3.10. Co-Infection and Co-Infection Index (Ic) 

Because *Y. enterocolitica* was not investigated in all animals, it was not considered for co-infection analysis. From the 28 positive animals, 8 (28.6%) were infected with just 1 investigated pathogen, 12 (39.3%) hosted 2 pathogens simultaneously, 4 (17.9%) hosted 3 pathogens, 3 (10.7%) hosted 4, and 1 animal (3.6%) hosted 5 pathogens (mupirocin-resistant *Staphylococcus* sp., non-pathogenic *Y. enterocolitica*, *L. monocytogenes*, *P. aeruginosa*, and *Vibrio* sp.). The most common combination (Table 5) was *Pseudomonas* spp. and *Salmonella* spp. (19.4% of all bacteria combinations), followed by *Staphylococcus* spp. associated with *Salmonella* spp. (13.9%), *Mycobacterium* spp. with *Salmonella* spp. or *Pseudomonas* spp. (8.3%), and, lastly, *Pseudomonas* spp. with *Staphylococcus* spp. (8.3%).

A positive co-infection index added to a chi-square test with a *p*-value less than 0.05 indicates a synergic relationship between the two bacteria present in the same animal. All co-infection indexes and statistical test results are shown in Table 6.

According to the data obtained, there is a strong correlation between *S.* Typhi infection and *the E. coli-*carrying *stx*_1_ gene, while *L. monocytogenes* is related to the development of *Staphylococcus* spp.

## 4. Discussion 

### 4.1. Campylobacter spp.

*Campylobacter* spp. are common zoonotic pathogens with relevance both in human and animal health, with rising incidence due to their ability to infect a wide variety of different species, their adaptability to new habitats, and the development of strains resistant to routine antibiotics [21]. In humans, the species that causes disease with more frequency is *C. jejuni*, followed by *C. coli*, even though more than 10 species have been identified. In reptiles, the predominant species are *C. iguaniorum*, *C. geochelonis*, and *C. fetus* subsp. *testudinum* [22,23]. Among the two positive isolates for *Campylobacter* spp. identified in this study, one corresponds to *C. lari*, a species rarely found in humans with campylobacteriosis [24], while the second species could not be identified, but it could be *C. iguaniorum* or *C. geochelonis*, both usually detected in reptiles with no data reported of causing disease in humans, although their discovery is recent and more data are needed to discard the human infection [25,26]. 

After an exhaustive bibliographic search, no reference was found regarding *C. lari* as a pathogen or commensal flora in reptiles; therefore, this could be the first case reported. *Campylobacter lari* is rather found in coast-related animals like shorebirds or seafood [27], so its presence in these animals could be explained by the fact that the chameleon population on Gran Canaria is located close to the coast where numerous marine animals live, especially birds like seagulls or shearwaters [28]. The cases of human infection with *C. lari* are not as frequent as *C. jejuni* or *C. coli*, although bacteremia cases have been reported [27,29,30] both in immunocompromised and immunocompetent individuals, which could risk the patient’s life.

### 4.2. Escherichia coli Virulence Genes (stx1, stx2, and eae) 

*Escherichia coli* presence, as well as the determination of its virulence factors, like its capacity to produce toxins or its antibiotic or heat resistance, has been highly studied around the world with different samples: humans, animals, food, water, or the environment [31,32,33]. Nevertheless, few works have been performed on cold-blood animals since cattle and their derived products are the principal *E. coli* reservoir associated with human disease [34]. In reptiles, the *E. coli* prevalence and virulence are lower, but their role as reservoirs capable of maintaining the transmission of this bacterium cannot be discarded [35,36].

In this study, the number of *E. coli*-carrying *stx*_1_, *stx*_2_, and *eae* virulence genes was similar to other studies, such as Bautista Trujillo et al. [37], in which 27 out of 240 (11.25%) captive green iguanas (*Iguana iguana*) from Mexico were positive for these genes, with a higher prevalence of the *stx*_1_ gene (10.0%) than the *stx*_2_ (0.4%) and *eae* (0.83%) genes. In our study, the difference was not as remarkable (5.0% of *stx*_1_ gene prevalence against 2.5% of the *eae* gene), and *stx*_2_ gene amplification did not happen. On the other hand, neither in Dec et al. [35] work on reptiles from Poland nor Martínez et al. [38] work on ocellated lizards (*Timon lepidus*) from Spain were the *stx*_1_, *stx*_2_, or *eae* genes found. 

Both the *stx*_1_ and *stx*_2_ genes are associated with severe symptoms like hemolytic uremic syndrome; these vary in the target organ and can cause different pathologies depending on the animal host; for example, in humans, *stx*_2_ gene expression is more often related to clinical complications than *stx*_1_ [39]. The intimin protein codified by the *eae* gene is involved in bacterial adherence to the intestinal epithelium and may or may not be present simultaneously with the *stx*_1_ and *stx*_2_ genes [40]. The large number of *E. coli* virulence genes, also divided into many subtypes with distinct particularities such as trophism for different tissues, makes *stx*_1_, *stx*_2_, and *eae* gene identification alone insufficient to determine the pathogenicity of the strain precisely [41]. 

### 4.3. Listeria monocytogenes

Listeriosis is an infrequent outbreak-associated disease that can cause severe health problems in humans and animals such as meningoencephalitis, septicemia, or fetal development failure if the infection affects pregnant women. It is transmitted through the ingestion of contaminated food, fundamentally meat [42,43]. 

Most studies regarding *L. monocytogenes* in animals are focused on mammals (mostly cattle) and birds, as well as food production chains [44,45]. Few authors have carried out investigations to analyze the presence of this bacterium in reptiles. Weber et al. identified 5/30 (16.7%) and 1/76 (1.3%) positive isolates of *L. monocytogenes* in turtles and snakes, respectively, in 1993, and 5/35 (14.29%) positive turtles in 1995 [46,47]; in both cases, the analyzed animals were pets. Similar data were published by Chen et al. showing a prevalence of 12% (2/17) in wild turtles [48]; however, the prevalence obtained by Nowakiewicz et al. in *Emys orbicularis* turtles was just 1.5% (2/130) [49]. Compared to those works, this study holds an intermediate place since the prevalence obtained was 5% (2/40). Equally, to humans, reptile listeriosis can be fatal and cases have been reported affecting bearded dragons (*Pogona vitticeps*) [50,51] and marine turtles (*Caretta caretta*) [52].

The ability of *Listeria monocytogenes* to survive in a wide range of conditions and the studies that evidence its presence in pet reptiles, like veiled chameleons, implies a potential risk of infection to humans, especially pet owners, and animal handlers.

### 4.4. Mycobacterium spp.

*Mycobacterium* spp. infection in reptiles is not as frequent as in mammals or birds, and most of the species involved belong to the atypical mycobacteria group, which rarely affects human beings, but its prevalence is globally increasing [53,54]. Commonly, non-tuberculous mycobacteria cause granulomas in different tissues of reptiles [55], even though no injuries were observed during the necropsies. Because of this, most of the studies are case reports of reptiles with granulomatosis [56,57,58], and few of the works found use samples of healthy animals. 

Isolates from five different individuals were positive for *Mycobacterium* spp. (12.5%), having similar results as Ebani et al. [59] in healthy reptiles from Italy. In that study, a significant difference was also found between the prevalence in snakes, saurians, and chelonians, being much higher in the first ones (72.2%, 9.7%, and 15.5%, respectively).

*rpoB* gene amplification occurred in three bacterial isolates, indicating an infection with *M. microti* or non-tuberculous bacteria. It probably corresponds to mycobacteria from the outer tuberculous complex, more common in reptiles such as *Mycobacterium chelonae* or *Mycobacterium marinum* [60], with one human reptile-related case reported concerning the second one [61]. The other two samples were positive for *M. bovis*, a zoonotic tuberculous bacterium with relevance in human health [62], but it cannot be confirmed if *M. bovis* can develop inside veiled chameleons or if they are just carriers, similar to *Mycobacterium tuberculosis*, whose reservoir are human beings, but have been identified in a huge variety of animals [63].

### 4.5. Pseudomonas spp.

Bacteria of the *Pseudomonas* genus are widely distributed opportunistic pathogens of humans and animals. Of all of them, *P. aeruginosa* is the species most related to human disease, being responsible for severe nosocomial infections in patients with burns or cystic fibrosis [64]. Moreover, this microorganism usually expresses antibiotic-resistant genes, making treatment ineffective in these cases [65,66]. In reptiles, cutaneous pathology is common, with ulcers or dermatitis [67,68], but other tissues may also be harmed [69]. 

In this study, 32.5% (13/40) of the sampled chameleons were infected with *Pseudomonas* spp. and 20% (8/40) with *P. aeruginosa*. These results are similar to Muñoz-Ibarra et al. in reptiles [66] (order Testudines and Squamata) from the Iberian Peninsula, who obtained a prevalence of 23.2% for *Pseudomonas* spp. and 18.0% for *P. aeruginosa*. Equally, Cristina et al. found 11.6% positive isolates of *P. aeruginosa* in snakes, lizards, and turtles from Romania [70]. The prevalence was lower in reptiles from Italy, where Ebani et al. [65] identified 22 (10.1%) positive samples for *Pseudomonas* spp. out of 218 animals among saurian, ophidians, and chelonids, with 4.1% of *P. aeruginosa*. In contrast, Andrea Sala et al. [71] described a *P. aeruginosa* prevalence of 59.9% in snakes from Italy, marking the variability of *Pseudomonas* spp. presence between different types of reptiles. All of these works were carried out with samples from captive animals or pets; hence, different results could be obtained from wild fauna. Colinon et al. [72] analyzed samples from captive and wild animals looking for the presence of *P. aeruginosa* and found prevalences of 87% in captive animals and 12% in wild ones; nevertheless, this difference could be due to different factors like the animal species, the geographical location, and/or human contact.

Apart from the clinical signs described in reptiles, *Pseudomonas* spp. can be found as regular oral and fecal microbiota, making them capable of transmitting these bacteria to animal handlers by bites or contact with feces [72]. 

### 4.6. Salmonella spp.

Reptiles are well-known carriers of *Salmonella* spp. in their gastrointestinal tract and numerous serotypes capable of causing disease, either in humans or reptiles, have been identified; in fact, there are many reports of reptile-associated salmonellosis from all around the world [73]. Most of the serotypes belong to *Salmonella enterica* subsp. *Enterica*, which is responsible for 99% of human infections; *S.* Typhi causes typhoid fever and is found typically in humans, but also produces no harm, whereas *S.* Typhimurium and *S.* Enteritidis cause gastrointestinal symptomatology in birds and mammals, including humans beings [74,75].

*Salmonella* spp. prevalence is highly variable between reptiles; for example, in their investigations, Bjelland et al. [73], Cota et al. [76], and Maja Lukac et al. [77] agree that chelonians are infected in a smaller proportion compared to saurian and ophidians. Moreover, in Merkevičienė et al. [78] study, a significant difference was observed between the prevalence in wild and domestic reptiles, being higher in the last ones (18.2% and 61.3%, respectively). The 40.0% prevalence for *Salmonella* spp. obtained in this study is comparable with Hydeskov et al. [79] in reptiles from Denmark (35.0%) and Corrente et al. in Italy with 50.5%, where all (5/5) *C. calyptratus* were positive [80]. In Gran Canaria, Monzón Moreno et al. reported a 100% (17/17) *Salmonella* spp. infection in the endemic lizard *Gallotia stehlini* [81], and Santana-Hernández et al. reported a prevalence of 20.5% in California Kingsnake (*L. californiae*) on the same island [82]. 

The presence of zoonotic *S.* Typhi and *S.* Typhimurium serotypes in the investigated animals demonstrates the transmission risk to people in close contact with chameleons, like animal handlers, with the possibility of developing severe disease. In addition, *Salmonella* species or serotypes not yet identified with the PCR assay employed could also be problematic and cause harm to humans or other animals.

### 4.7. Staphylococcus spp.

*Staphylococcus* is a broad bacterial genus forming part of normal skin and mucosa microflora of animals, birds, and reptiles [83]. In humans, the most relevant species is *S. aureus* because is responsible for many nosocomial infections that can risk a patient’s life, especially in the case of antibiotic-resistant strains. This bacterium can also harm animals, together with the *Staphylococcus hyicus* and *Staphylococcus intermedius* group, even though these have a limited host range compared to *S. aureus* [84,85]. None of the sampled animals were positive for *S. aureus*, which matches with other studies in reptiles such as Espinosa-Gongora et al. [86], in which no bacteria were isolated in any of the 21 samples examined from multiple reptile species, or Cristina et al. [70] work, with a *S. aureus* prevalence of 2.5% (1/43). 

In our study, two *Staphylococcus* spp. isolates were positive for the methicillin-resistant gene and the other two for the mupirocin-resistant gene, and also one juvenile individual was positive for both antibiotic-resistant genes. This involves difficulty for treatment, even though the species could not be identified in the PCR assay and zoonotic transmission may not occur.

The only species identified was *S. hominis* in just one isolate; this microorganism is frequently found in human skin along with *S. epidermidis* and is a rare health problem; on the contrary, its protective role against opportunistic cutaneous infections has been studied [87,88]. However, there have been cases reported of severe infections, especially after prosthetic surgery, due to their ability to form biofilms [89,90]. In animals, its presence is uncommon and probably due to human contact [91,92,93].

### 4.8. Vibrio spp.

Around 80 species of *Vibrio* are known, of which 12 are capable of affecting humans, commonly *V. cholerae*, *V. parahemolyticus*, and *V. vulnificus*. They are water-associated bacteria that can grow under different temperature, pH, and salt conditions and cause human disease after eating contaminated marine-based food, particularly seafood [94], causing gastrointestinal problems with symptoms depending on the involved bacteria [95,96]. 

Few studies have been conducted about the presence of *Vibrio* spp. in non-marine animals and their role in spreading these bacteria is not clear, and vibriosis can occur possibly by the ingestion of reptile-origin food [97] or by bites [98]. *Vibrio cholerae* have been found both in the skin and gastrointestinal tract of soft-shell turtles [99]; hence, it is probable that the transmission to humans occurred through aquatic reptiles used as pets, but more studies are needed to prove this possibility. In Tenerife, the largest island of the Canarian archipelago, *Vibrio cholerae* was identified in feces from *Anolis* sp., another introduced reptile [100]. 

In terrestrial reptiles, such as veiled chameleons, *Vibrio* spp. infections do not seem to be frequent and their origins may be the water or food; consequently, *Vibrio* spp. determination in animals should be always attached to the analysis of nearly water masses. The absence in this study of species found commonly involved in human vibriosis does not discard the possibility of them causing disease since they are not the only ones capable of affecting humans, even though their low prevalence obtained in this work (2.5%) makes infections difficult.

### 4.9. Yersinia enterocolitica

*Yersinia enterocolitica* is an important zoonotic agent, which causes fever, gastrointestinal symptoms, and lymphadenopathy, and enters the organism mainly through pork meat but also milk and water. Direct person-to-person contact or via animals and their faces transmission routes have been also described [101].

Few studies have been carried out looking for *Y. enterocolitica* in reptiles because they are typically associated with mammals, with pigs and boars as the main reservoirs [102]. The high prevalence of *Y. enterocolitica* (52.4%) found in the veiled chameleons studied greatly contrasts with previous works such as Shayegani et al. [103], in which none of the reptile samples were positive for this bacterium, or Nowakiewicz et al. [49] with only 2 out of 130 turtles infected. It must be emphasized that the *ail* gene virulence factor associated with adhesion and invasion of the intestinal wall [104] was only identified in one of the eleven positive isolates for *Y. enterocolitica*; for this reason, the probability of this high prevalence for producing disease in humans is lower. 

To discover why this prevalence is elevated, other animals in contact with the chameleons must be analyzed as well as the surrounding water supplies or diet to establish a clear cause. The insect-based diet of the veiled chameleons could be the reason for the difference between the reported prevalences and the one obtained in this work, because the invertebrates could be hosting the bacteria from scavenging or because of the ingestion of infected feces of mammals. This phenomenon does not occur in other reptiles such as turtles or snakes.

### 4.10. Summary

In the *C. calyptratus* investigated population, the most prevalent bacteria were *Y. enterocolitica*, followed by *Salmonella* spp. and *Pseudomonas* spp, with *Y. enterocolitica*, *Salmonella* spp., and *E. coli* being well-known zoonotic species. Lower prevalences were found in the previous few studies conducted looking for *Y. enterocolitica* in reptiles, while more than half of the sampled chameleons were infected with this bacterium in this study. The data obtained around the other three bacteria are quite similar to the other several works carried out.

Multi-infection in the same individual is usual in nature due to exposure to different pathogens, not only to bacteria but to viruses, fungi, or parasites, which coexist in the environment or within the food [105]. In this case, half (20/40) of the studied animals were infected with more than 1 microorganism, 8 of them with 3 or more simultaneously. Moreover, significant relationships were observed between different bacteria species, indicating that the presence of one of them facilitates the infection of another one; highlights: *S.* Typhi with *E. coli* carrying the *stx*_1_ gene and *L. monocytogenes* with *Staphylococcus* sp. Further investigation is needed to understand the mechanisms that ease these co-infections.

Even though this work did identify bacteria in reptiles not previously reported, *C. lari*, *M. bovis*, and *S.* Typhi, the role of the veiled chameleons in their transmission is not clear, and more studies must be conducted to confirm if those bacteria are capable of development within these animals or they have been accidentally found after having been ingested along with water or food or through contact with other animals or the environment. 

The presence of all the pathogens identified in *C. calyptratus* from Gran Canaria, some of them with well-known zoonotic activity, could be a health risk to both human and animal populations who contact them, mainly if correct handling procedures are not applied. 

## 5. Conclusions

An alien *Chamaeleo calyptratus* population studied in the Canary Islands (Spain) harbors various bacteria with importance to public health. Taking account of the zoonotic importance of many of the pathogens detected, handling *C. calyptratus* from Gran Canaria supposes a risk to human health. *Yersinia enterocolitica*, pathogenic *Escherichia coli*, and *Salmonella* spp. were detected, all of them being species with recognized zoonotic potential.

Veiled chameleons could also transmit pathogens to native animals, including endemic species, and/or spread them to the environment, so their possible role against biodiversity should be taken into account. Considering the human and veterinary health importance of the present results, even by analyzing a limited number of samples, more studies are needed with larger sample sizes to better understand the epidemiology of the pathogenic bacteria present in the invasive *C. calyptratus* in the Canary Islands.

## Figures and Tables

**Table 1:** Percentage of isolated bacteria from *Chamaeleo calyptratus* from Gran Canaria (Canary Islands, Spain).

Bacteria	No. of Positive Isolates (n)	Prevalence (%)
*Campylobacter* spp.	2 (40)	5.0
*Escherichia coli* (*eae*, *stx*1, and *stx*2 genes)	3 (40)	7.5
*Listeria monocytogenes*	2 (40)	5.0
*Mycobacterium* spp.	5 (40)	12.5
*Pseudomonas* spp.	13 (40)	32.5
*Salmonella* spp.	16 (40)	40.0
*Staphylococcus* spp.	7 (40)	17.5
*Vibrio* spp.	1 (40)	2.5
*Yersinia enterocolitica*	11 (21)	52.4

**Table 2:** Prevalence of *Pseudomonas* spp. in *Chamaeleo calyptratus* from Gran Canaria (Canary Islands, Spain).

Bacteria	Positive Males (Prevalence) n = 11	Positive Females (Prevalence) n = 21	Positive Juveniles (Prevalence) n = 4	Total Positive Individuals (Prevalence) N = 40
*Pseudomonas* spp.	5 (45.5%)	7 (33.3%)	1 (25.0%)	13 (32.5%)
*Pseudomonas aeruginosa*	3 (27.3%)	5 (23.8%)	0	8 (20%)

**Table 3:** Prevalence of *Salmonella* spp. in *Chamaeleo calyptratus* from Gran Canaria (Canary Islands, Spain).

Bacteria	Positive Males (Prevalence) n = 11	Positive Females (Prevalence) n = 21	Indetermined Sex (Prevalence) n = 4	Positive Juveniles (Prevalence) n = 4	Total Positive Individuals (Prevalence) N = 40
*Salmonella* spp.	4 (36.4%)	9 (42.9%)	1 (25.0%)	2 (50.0%)	15 (37.5%)
*S.* Typhi	0	3 (14.3%)	0	0	3 (7.5%)
*S.* Typhimurium	1 (9.1%)	2 (9.52%)	0	0	3 (7.5%)
*S.* Enteritidis	0	0	0	0	0

**Table 4:** Prevalence of *Staphylococcus* spp. and antibiotic-resistant genes in *Chamaeleo calyptratus* from Gran Canaria (Canary Islands, Spain).

Bacteria	Positive Males (Prevalence) n = 11	Positive Females (Prevalence) n = 21	Positive Juveniles (Prevalence) n = 4	Total Positive Individuals (Prevalence) N = 40
*Staphylococcus* spp.	1 (9.1%)	5 (23.8%)	1 (25.0%)	7 (17.5%)
*S. hominis*	0	1 (4.8%)	0	1 (2.5%)
Methicillin resistance gene	1 (9.1%)	2 (9.5%)	1 (25.0%)	4 (10.0%)
Mupirocin resistance gene	0	2 (9.5%)	1 (25.0%)	3 (7.5%)

**Table 5:** Percentage of co-infections in *Chamaeleo calyptratus* from Gran Canaria (Canary Islands, Spain).

No. of Animals with Co-Infections	Identified Bacteria/Virulence Genes	Percentage% of Co-Infections (n = 36)
7	*Pseudomonas* spp. + *Salmonella* spp.	19.4
5	*Salmonella* spp. + *Staphylococcus* spp.	13.9
3	*Mycobacterium* spp. + *Pseudomonas* spp.	8.3
3	*Mycobacterium* spp. + *Salmonella* spp.	8.3
3	*Pseudomonas* spp. + *Staphylococcus* spp.	8.3
2	*L. monocytogenes* + *Staphylococcus* spp.	5.6
2	*S.* Typhi + *stx*_1_ gene	5.6
1	*C. lari* + *Mycobacterium* spp.	2.8
1	*eae* gene + *Mycobacterium* spp.	2.8
1	*eae* gene + *P. aeruginosa*	2.8
1	*eae* gene + *S.* Typhimurium	2.8
1	*L. monocytogenes* + *P. aeruginosa*	2.8
1	*L. monocytogenes* + *Vibrio* spp.	2.8
1	*Mycobacterium* spp. + methicillin-resistant *Staphylococcus* sp.	2.8
1	*P. aeruginosa* + *Vibrio* spp.	2.8
1	*S. hominis* + *P. aeruginosa*	2.8
1	methicillin-resistant *Staphylococcus* sp. + *stx*_1_ gene	2.8

**Table 6:** Co-infection index for the different co-infections obtained from *Chamaeleo calyptratus* from Gran Canaria (Canary Islands, Spain).

Identified Bacteria/Virulence Genes	Co-Infection Index (Ic)	*p*-Value
*Pseudomonas* spp. + *Salmonella* spp.	8.2	0.215
*Salmonella* spp + *Staphylococcus* spp.	17.8	0.094
*Mycobacterium* spp. + *Pseudomonas* spp.	9.2	0.307
*Mycobacterium* spp. + *Salmonella* spp.	5.6	0.373
*Pseudomonas* spp. + *Staphylococcus* spp.	4.3	0.662
***L. monocytogenes* + *Staphylococcus* spp.**	**21.4**	**0.027**
***S.* Typhi + *stx*_1_ gene**	**61.7**	**0.004**
*C. lari* + *Mycobacterium* spp.	17.5	0.125
*eae* gene + *Mycobacterium* spp.	17.5	0.125
*eae* gene + *P. aeruginosa*	10.0	0.200
*eae* gene + *S.* Typhimurium	30.8	0.075
*L.monocytogenes* + *P. aeruginosa*	6.7	0.364
*L.monocytogenes* + *Vibrio* spp.	47.5	0.050
*Mycobacterium* spp. + methicillin-resistant *Staphylococcus* sp.	6.3	0.427
*P. aeruginosa* + *Vibrio* spp.	10.0	0.200
*S. hominis* + *P. aeruginosa*	10.0	0.200
Methicillin-resistant *Staphylococcus* sp. + *stx*_1_ gene	16.0	0.192

Bold type: significant differences between the number of co-infections obtained and expected.

## Data Availability

All data obtained are included within the article.
